# Computational analysis predicts the Kaposi's sarcoma-associated herpesvirus tegument protein ORF63 to be alpha helical

**DOI:** 10.1002/prot.24097

**Published:** 2012-04-19

**Authors:** Joseph P Boyle, Tom P Monie

**Affiliations:** Department of Biochemistry, University of CambridgeCambridge CB2 1GA, United Kingdom

**Keywords:** innate immunity, NOD-like receptors, NLR, immune evasion, herpesviruses, protein homology modeling, protein structure prediction, virus, TLR

## Abstract

The innate immune response provides our first line of defence against infection. Over the course of evolution, pathogens have evolved numerous strategies to either avoid activating or to limit the effectiveness of the innate immune system. The Kaposi's sarcoma-associated herpesvirus (KSHV) contains tegument proteins in the virion that contribute to immune evasion and aid the establishment of viral infection. For example, the KSHV tegument protein ORF63 modulates inflammasome activation to inhibit the innate immune response against the virus. Understanding the likely structure of proteins involved in immune evasion enables potential mechanisms of action to be proposed. To understand more fully how ORF63 modulates the innate immune system we have utilized widely available bioinformatics tools to analyze the primary protein sequence of ORF63 and to predict its secondary and tertiary structure. We found that ORF63 is predicted to be almost entirely alpha-helical and may possess similarity to HEAT repeat containing proteins. Consequently, ORF63 is unlikely to be a viral homolog of the NLR protein family. ORF63 may inhibit the innate immune response by flexibly interacting with its target protein and inhibiting the recruitment of protein co-factors and/or conformational changes required for immune signaling.

## INTRODUCTION

Viral infection activates multiple arms of the innate immune response and many pattern recognition receptors (PRRs) are involved.^1^, ^2^ These include: the Toll-like receptor family; members of the RIG (retinoic acid inducible gene)-I-like receptor (RLR) family; and other cytoplasmic receptors such as the IFI16 [Interferon (IFN) gamma-inducible protein 16] family, DExD/H box RNA helicases, DNA-dependent activator of IFN-regulatory factors (DAI), and RNA Pol III.^3^ Recently, the nucleotide-binding leucine-rich repeat containing (NLR) family have also been implicated in the cellular response to viral infection. For example, the NLRP3 inflammasome is activated by adenovirus double-stranded DNA^4^ and influenza virus^5^; whereas NOD2 has been reported to be stimulated by viral ssRNA.^6^ Evasion of the host innate immune response is critical for the survival and pathogenesis of many viruses and multiple evasion strategies are used.^7^ For instance, Hepatitis C Virus NS3-4A protease inactivates RLR signaling by cleaving the adaptor MAVS (mitochondrial antiviral signaling protein)^8^ and the human cytomegalovirus tegument protein UL83 interferes with the induction of IFN-stimulated genes.^9^

The gamma herpesvirus Kaposi's sarcoma-associated herpesvirus (KSHV) is associated with neoplastic diseases including Kaposi's Sarcoma, primary effusion lymphoma, and multicentric Castleman's disease. It produces a wide range of immunomodulatory and immune evasion proteins that interfere with multiple facets of the host immune response, including: IFN signaling, complement activation, apoptosis, autophagy, cytotoxic T cells, Natural Killer cells, and the chemokine response.^10^ Located between the KSHV viral capsid and envelope is a protein-rich region known as the tegument. The precise number of KSHV tegument proteins is uncertain. Purification of extracellular virions identified six different viral proteins within the tegument: ORF21, ORF33, ORF45, ORF63, ORF64, and ORF75.^11^ Although these tegument proteins still require full characterization, our understanding of their function is improving. Direct studies with KSHV tegument proteins and inference from studies on homologous proteins in other herpesviruses have suggested roles for tegument proteins in viral egress, assembly, envelopment, immune modulation, and as thymidine kinases and deubiquitinases.^12^–^20^

Recently, further insight into the role of both KSHV ORF63 and ORF64 tegument proteins in immune evasion has been provided.^13^, ^20^ KSHV ORF64 interferes with RIG-I mediated IFN production through deubiqutination of RIG-I.^20^ In contrast, KSHV ORF63 has been reported to be a viral NLR-homolog that can inhibit inflammasome activation in a manner that enhances viral reactivation and replication.^13^ Inflammasome activation is a key immune response resulting in the activation of caspase-1 and subsequent processing and secretion of Interleukin (IL)-1β and IL-18. In many instances inflammasome activation ultimately leads to caspase-1 dependent cell death through the process of pyroptosis. Multiple pattern recognition receptors such as NLRP3, NLRP1, NLRC4 and NAIP, and AIM2 (Absent in melanoma 2), are capable of forming inflammasomes.^21^ Inhibition of inflammasome function is an important and widely used immune evasion strategy of both viruses and bacteria. For example, poxviruses produce Pyrin only protein (POP)-like proteins that interfere with inflammasome assembly, whereas the influenza virus NS1 protein disrupts caspase-1 activation^22^ and the IFN response.^23^

We have used bioinformatics, computational modeling, and comparative sequence analysis to investigate the primary protein sequence and predict the secondary and tertiary organization of KSHV ORF63 to help understand its mode of action in relation to inflammasome activation. In this article we report that KSHV ORF63 is most likely to possess a herpesvirus U30 domain and possess a protein structure dominated by alpha helices. These attributes are conserved in a range of homologous herpesvirus tegument proteins. Potential tertiary structure homology was identified with the Huntingtin-elongation-A subunit-TOR (HEAT) repeat containing protein Cullin-associated NEDD8-dissociated protein 1 (Cand1). Our analysis suggests that although KSHV ORF63 disrupts inflammasome signaling it is not a viral-NLR homolog, as has been previously proposed.^13^

## MATERIALS AND METHODS

### Protein sequences

KSHV ORF63 and human NLRP1 protein sequences were extracted from the NCBI protein database using the accession numbers YP_001129420.1 and Q9C000, respectively. The accession numbers of KSHV ORF63 homologs identified by BLASTp are listed in Table I.

**Table I:** Significant BLASTp Hits from the Nonredundant Protein Database Showing Homology with KSHV ORF63

Protein	Accession	*E* value
Macacine herpesvirus 5 tegument protein	NP_570811.1	0.0
Rhesus monkey rhadinovirus H26-95 tegument protein	AAF60049.1	0.0
Macaca fuscata rhadinovirus JM123	AAT00100.1	0.0
Bovine herpesvirus 4 tegument protein	NP_076555.1	8e-144
Saimiriine herpesvirus 2 hypothetical protein SaHV2gp64	NP_040265.1	3e-127
Saimiriine herpesvirus 2 hypothetical protein	CAC84360.1	9e-126
Ateline herpesvirus 3 hypothetical protein AtHV3gp60	NP_048034.1	1e-119
Equid herpesvirus 2 tegument protein UL37	NP_042660.1	4e-106
Alcelaphine herpesvirus 1 tegument protein UL37	NP_065562.1	1e-94
Ovine herpesvirus 2 ORF63	YP_438187.1	1e-89
Ovine herpesvirus 2 tegument protein-like protein	ABB22282.1	1e-89
Murid herpesvirus 4 tegument protein	AAB66416.1	2e-58
Murid herpesvirus 4 tegument protein UL37	NP_044901.2	3e-58
Wood mouse herpesvirus tegument protein UL37	ACY41133.1	2e-54
Rodent herpesvirus Peru tegument protein	YP_004207899.1	2e-48
Callitrichine herpesvirus 3 ORF57	NP_733911.1	1e-44
Human herpesvirus 4 BOLF1	Q3KSU7.1	1e-26
Human herpesvirus 4 type 2 BOLF1	YP_001129450.1	8e-26
Human herpesvirus 4 strain B95-8 BOLF1	YP_401653.1	3e-25
Macacine herpesvirus type 4 BOLF1	YP_067951.1	4e-24

### Computational analysis of protein sequences

Protein sequences were analyzed by a variety of publicly available internet-based bioinformatics programmes. Unless stated in the text the default settings were used for the analysis. Conserved domains were identified using the NCBI Conserved Domains Database^24^ and BLASTp algorithms. Protein alignments were generated using CLUSTALW2 and BLASTp. Homologous proteins were identified using BLASTp, position-specific iterated (PSI)-BLAST, FUGUE,^25^ and GenThreader.^26^ Searches with BLASTp and PSI-BLAST used the nonredundant protein database and hence searched against protein sequences from all organisms. Secondary structure predictions were made using PsiPred.^27^, ^28^ Pattern specific profiling and searching was performed using PATTINPROT via the Network Protein Sequence Analysis server.^29^ Transmembrane helix predictions were made using the TMHMM Server v 2.0.^30^

## RESULTS

### KSHV ORF63 contains a herpesvirus U30 domain

**Figure 1:**
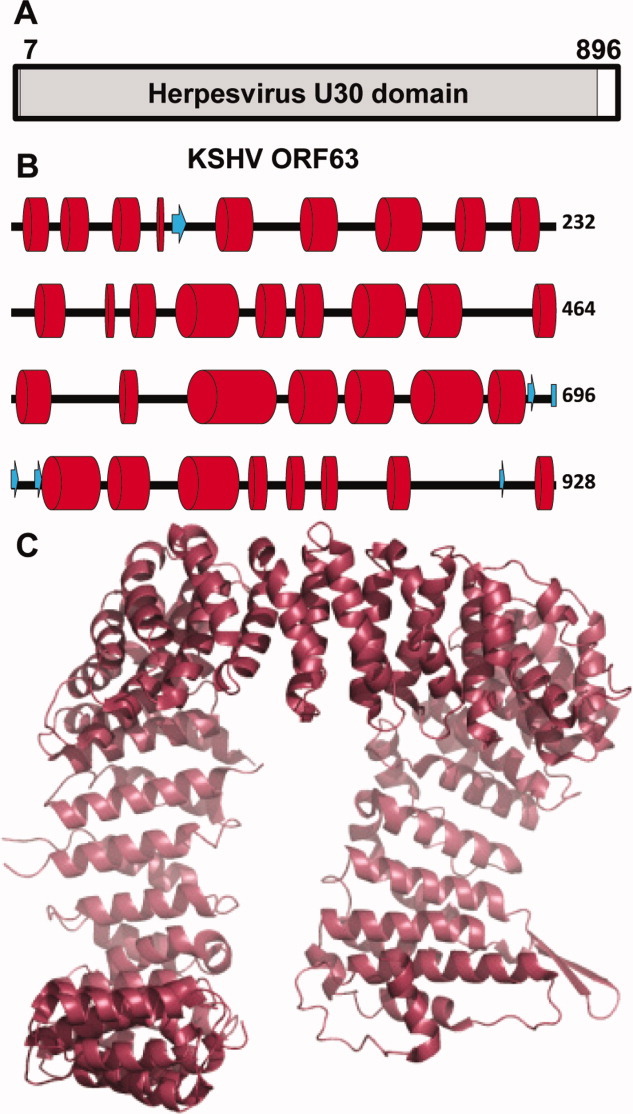
ORF63 is predicted to be predominantly alpha helical. **A:** The only conserved domain reported for KSHV ORF63 is the Herpesvirus U30 domain. **B:** Secondary structure prediction using PSIPRED suggests that KSHV ORF63 is predominantly alpha helical. Alpha helices are shown as red barrels, beta sheets as blue arrows. **C:** Tertiary structure of the most significant homologous protein to KSHV ORF63 identified by FUGUE and GenThreader – the HEAT repeat containing protein Cand1 (pdb 1u6g). Only the Cand1 component of the Cand1-Cul1-Roc1 complex is shown.^31^

An early step towards understanding protein function is to identify the potential protein domains and folds present in the polypeptide. We used KSHV ORF63 (Accession number YP_001129420.1) to search the NCBI (National Center for Biotechnology Information) Conserved Domains database.^24^ The only significant and recognizable conserved domain reported in full-length KSHV ORF63 was the herpesvirus tegument protein U30 domain (pfam ID: pf04523) [Fig. 1(A)]. This domain has not been found to date in non-herpesvirus proteins suggesting a specific functional role in the herpesvirus replication cycle.

To extend our search for conserved domains, we performed a BLASTp search for homologous proteins to KSHV ORF63. Twenty homologous proteins were identified, all of which possessed highly significant *E* values (Table I). All these proteins were of herpesvirus origin. To check for more distant homologs a PSI-BLAST search with either full-length KSHV ORF63, or the fragment (residues: 230–360) highlighted by Gregory and colleagues,^13^ was performed. The searches reached convergence after six and three iterations, respectively. The PSI-BLAST search identified neither NLRP1 nor any other NLR protein as a homolog of KSHV ORF63. KSHV ORF63 is therefore most likely to possess a herpesvirus U30 domain rather than any other currently characterized protein fold.

### The secondary structure of KSHV ORF63 is dominated by alpha helices

Proteins fold into domains possessing characteristic secondary structure patterns. Consequently, secondary structure predictions can provide insight into the potential protein architecture. The likely secondary structure of KSHV ORF63 was determined using PSIPRED^27^, ^28^ [Fig. 1(B)]. The predicted protein architecture is dominated by the presence of alpha helices connected with loops and coils [Fig. 1(B)]. Consistent with its function as a tegument protein none of the helices are predicted to be transmembrane spanning.

Strong clues about protein function and mechanisms of action can be provided through the identification of proteins with homologous tertiary structure. KSHV ORF63 has been previously reported to be a homolog of the NLR protein family based on the presence of short regions of similar sequence.^13^ We sought to extend these observations in the context of the whole protein and generate a global perspective on the tertiary structure of ORF63. The KSHV ORF63 primary sequence was submitted to the homology search servers Genthreader^26^ and Fugue.^25^ Both GenThreader and FUGUE returned, with high confidence (>95% confidence FUGUE; GenThreader *P* value <0.0001), the Cand1 component of the Cand1-Cul1-Roc1 complex as the most significant hit^31^ [PDB 1u6g; Fig. 1(C)]. Interestingly, Cand1 is 61% helical, lacks beta sheets, and forms an extended sinuous solenoid from 27 tandem HEAT repeats. Other homologs of KSHV ORF63 (Table I) also consistently returned hits of highly helical proteins including Cand1, the importin alpha re-exporter, and the HEAT-like repeat containing Exportin-1 and Exportin-5 (data not shown). A PSIBLAST search with KSHV ORF63 did not return HEAT repeat containing proteins within the threshold of detection.

### The predicted KSHV ORF63 secondary and tertiary structure is different to NLRP1

The results of the secondary and tertiary structure predictions are inconsistent with the assertion that ORF63 is a homolog of the NLR family member NLRP1.^13^ Our data identifying the presence of a conserved herpesvirus U30 domain, the strong alpha helical secondary structure, and the predicted tertiary homology to HEAT repeat containing proteins are indicative of an alternative structural composition and alternative evolutionary homology. To try and resolve this discrepancy, we took a closer look at the primary sequence of KSHV ORF63 and also compared the predicted secondary and tertiary structures of KSHV ORF63 and NLRP1.

**Figure 2:**
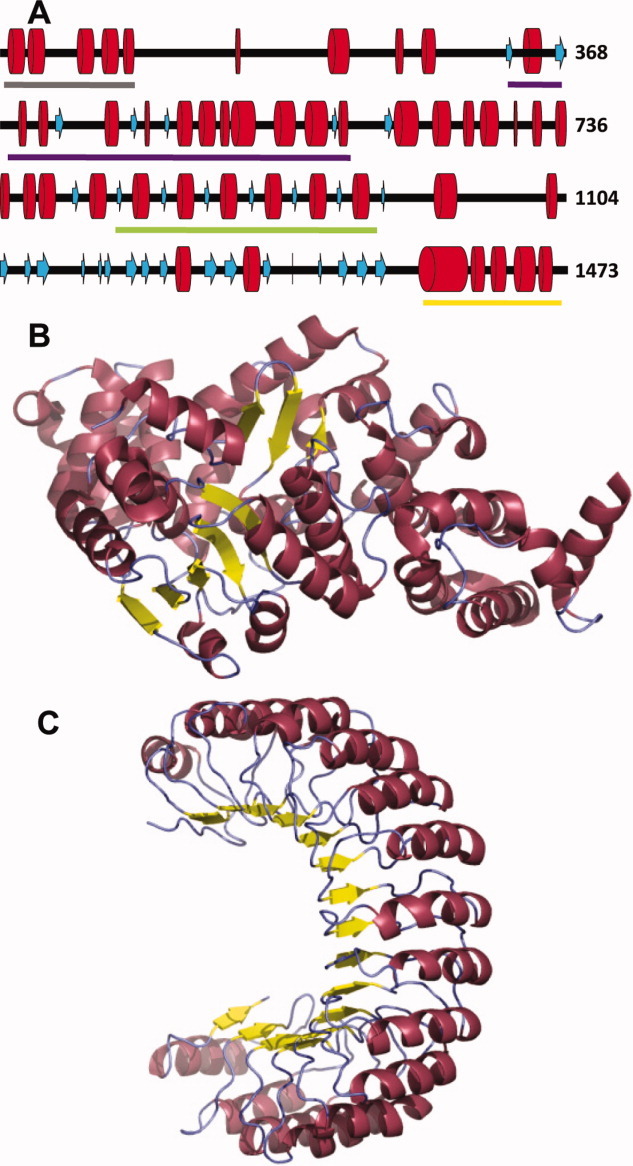
NLR proteins are multidomain. **A:** PSIPRED prediction of the NLRP1 secondary structure reveals both alpha helical and beta sheet components. Alpha helices are shown as red barrels, beta sheets as blue arrows. The major domains of NLRP1 are underlined: PYRIN domain (gray), Nucleotide binding and NACHT domain (purple), LRRs (green), and caspase activation and recruitment domain (yellow). The Function to Find Domain (FIIND) has not been annotated as its topology is poorly defined. It is however situated between the LRRs and caspase activation and recruitment domain. **B:** Apaf-1 nucleotide binding and NACHT domain (pdb 1z6t^32^). **C:** Ribonuclease inhibitor LRR domain (pdb 1dfj^33^). NLR proteins possess LRRs with a characteristic ribonuclease repeat. In both panels A and B alpha-helices are colored purple and beta-sheets yellow.

Topological predictions of NLRP1 highlight the multidomain nature of the NLR family of proteins and the presence of both alpha-helices and beta-sheets in these domains [Fig. 2(A)]. This is in contrast to the predominantly alpha-helical organization of KSHV ORF63 [Fig. 1(B)]. NLRP1 possesses a helical N-terminal Pyrin domain effector domain with a Death Domain family fold; a three layered alpha-beta sandwich conformation from the NBD; an alternating beta-sheet and alpha-helix or loop structure due to the presence of a curved solenoid LRR; a beta-sheet rich FIIND (function-to-find domain); and a C-terminal alpha-helical caspase activation and recruitment domain (CARD) [Fig. 2(A)]. With the exception of NLRP10, which lacks any LRRs; and NLRP1, which has additional C-terminal FIIND and CARD folds, NLR family members are tripartite in organization and end with a C-terminal LRR. NLRP1 and KSHV ORF63 are predicted to have diverse secondary structures [Figs. 1(B) and 2].

The NBD and LRR domains of the NLR proteins contain a mix of alpha-helices and beta-sheets. Although neither the NBD nor LRR domains of NLR proteins have to date had their structures solved, there are plenty of examples of these protein folds [Fig. 2(B,C)]. NLR proteins are predicted to share structural homology with the NBD of Apaf-1^34^ in which the core parallel beta sheets locate the motifs critical for nucleotide binding [Fig. 2(B)]. LRRs adopt a curved solenoid with a beta sheet concave surface and a more variable, but often helix- and loop-rich, convex surface [Fig. 2(C)].^35^ Neither of these motifs would be structurally compatible with the strongly helical KSHV ORF63 secondary structure prediction [Fig. 1(B)].

### The primary sequence of KSHV ORF63 does not support the presence of an NBD motif or an LRR domain

**Figure 3:**
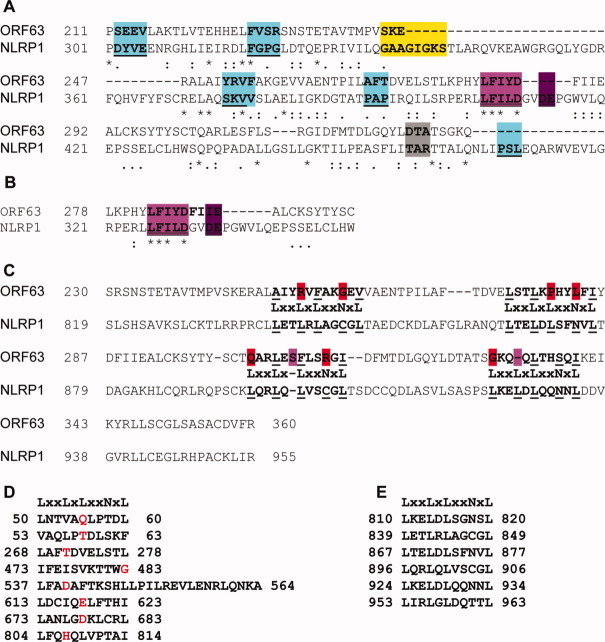
KSHV ORF63 lacks the key motifs required for possession of a nucleotide binding domain or leucine rich repeat. **A:** ClustalW2 alignment of KSHV ORF63 with the nucleotide binding domain of NLRP1. Key motifs and residues are in bold type; the Walker A motif is highlighted yellow; the Walker B motif is highlighted purple; the characteristic NLR “DE” motif is highlighted deep purple; the sensor 1 motif is highlighted gray; and residues predicted to be involved in protein oligomerization^34^ are highlighted cyan. **B:** Manually adjusted alignment around the Walker B region. Colors as in panel A. In both alignments conserved (*), conservatively substituted (:), and semi-conservatively substituted (.) are annotated. **C:** BLASTP alignment of ORF63 and NLRP1 (*E* = 0.0002, 27% identity). The Ribonuclease Inhibitor LRR consensus sequence (LxxLxLxxNxL where L = Leu, Val, Ile, Phe; N = Asn, Cys, Ser, Thr; x = any amino acid) has been added between the alignments for each LRR. LRR consensus regions are in bold type, with the key residues underlined. Sites in each LRR at which ORF63 differs from the consensus sequence are highlighted in red (substitution) or purple (insertion/deletion). Putative KSHV ORF63 **(D)** and human NLRP1 **(E)** LRR motifs identified by PROSCAN. Mismatches are highlighted in red. Searching was performed using the consensus sequence reported in panel C.

Given the difference in predicted structure between KSHV ORF63 and NLRP1 we looked more closely at the region of primary sequence reported to be homologous between the proteins. We reproduced the alignment of Gregory and colleagues^13^ between the NLRP1 NBD (accession number Q9C000) and KSHV ORF63 using ClustalW2 [Fig. 3(A)]. This alignment could not be reproduced using BLASTp. As outlined earlier (Table I), the use of BLASTp to search for homologous sequences, rather than align user-provided sequences, did not recover NLRP1 as a hit, significant, or otherwise. Manual analysis of the ClustalW2 NLRP1 NBD and KSHV ORF63 alignment [Fig. 3(A)] identified potential conservation of the residues forming the Walker B motif in KSHV ORF63. As with the NLR proteins, the second acidic residue^34^, ^36^ of the Walker B motif is absent. In the ClustalW2 alignment [Fig. 3(A)] KSHV ORF63 lacks conservation of the conserved DE motif found in NLRs immediately downstream of the Walker B motif, although manual adjustment of the alignment results in conservation of the second of these residues [Fig. 3(B)]. The Walker A and Sensor 1 motifs, critical for nucleotide binding and NBD function, are missing in KSHV ORF63 and there is poor conservation of the residues predicted to be involved in NBD mediated oligomerization [Fig. 3(A)].^34^ A PROSCAN^29^ analysis of KSHV ORF63 confirmed the absence of a Walker A motif anywhere within the protein.

A BLASTp search with full-length ORF63 does not result in any hits with NLR family members (Table I). However, BLASTp can be used to directly align KSHV ORF63 and human NLRP1 [Fig. 3(C)]^13^ with an *E* value of 0.0002. This discrepancy can be explained through the manner of *E* value calculation for the two methods. The *E* value created by BLASTp during the sequence alignment is not related to the *E* value calculated when searching for homologs in a sequence database. It is instead related to the length of the input sequence and hence carries a greater likelihood of reporting a significant similarity between two sequences that are in fact simply showing a chance similarity. Inspection of this alignment and that produced between KSHV ORF63 and the NLRP1 NBD [Fig. 3(A)] shows that they contain heavily overlapping regions of KSHV ORF63 [compare Fig. 3(A,C)] making the formation of two distinct protein domains from this region of KSHV ORF63 unlikely.

The BLASTp alignment is suggestive that KSHV ORF63 may contain LRR motifs as four of the NLRP1 LRRs are contained in the aligned region [Fig. 3(C)]. Each repeat in an LRR consists of a highly conserved segment and a variable segment. LRRs can be characterized into one of seven groups^37^ based on the conservation and spacing of the residues in the repeat.^38^ NLR proteins adopt a ribonuclease inhibitor conformation [Fig. 2(C)] and possess a highly conserved segment with the following consensus sequence: [LIVF]-X-X-[LIVF]-X-[LIVF]-X-X-[NSTC]-X-[LIVF] (where X is any amino acid).^37^, ^38^ Manual analysis of the NLRP1 LRR and KSHV ORF63 alignment reveals a lack of identity with the consensus LRR motif, which includes a complete lack of conservation of the asparagine ladder [Fig. 3(C)]. Multiple substitutions are observed in the consensus regions and two of the alignments require either an amino acid insertion or deletion, potentially compromising the integrity of the protein fold. Pattern profiling, using PROSCAN, against the LRR consensus motif did not identify any conserved LRR motifs in KSHV ORF63. Reducing the search stringency to 80% similarity identified seven unique, and one overlapping, motifs across the length of KSHV ORF63 [Fig. 3(D)]. Extending the consensus to include the Ribonuclease Inhibitor family variable segment retained only a single putative repeat beginning at residue L537 [Fig. 3(D)]. In contrast, performing the same analysis on isoform 1 of human NLRP1 identified six consecutive LRRs consistent with previous annotations^39^, ^40^ [Fig. 3(E)]. LRR domains are formed from between 2 and 45 contiguous repeats.^41^ Hence the spatial disposition of the putative motifs in KSHV ORF63 would not enable the formation of an LRR domain.

## DISCUSSION

The KSHV tegument protein ORF63 has been recently shown to act as an immune evasion protein and interfere with the activation of the NLRP1 inflammasome. To try and gain a better understanding of the molecular basis of KSHV ORF63 mediated inflammasome inhibition we performed a computational analysis of KSHV ORF63 to characterize potential structural and functional motifs. Our analysis indicates that the KSHV ORF63 protein is likely to be mainly alpha-helical in nature. We were unable to confirm the presence of either an NBD or an LRR in KSHV ORF63 making it unlikely that KSHV ORF63 is a viral homolog of the NLR family of proteins.

Homologous proteins have, by definition, evolved from a common ancestral protein. However, their divergent primary structures can be difficult to compare. For example, the death fold superfamily, which comprises the CARD, Pyrin Domain, Death Domain, and Death Effector Domain folds, shows as little as 10% sequence similarity between individual members.^42^ The overall folds of these proteins are however relatively conserved despite their differing sequences and their similar structures reflect their similar functions. Viruses tend to evolve rapidly. Hence, viral homologs of cellular proteins can display low sequence similarity while maintaining key aspects of secondary and tertiary structure. An example is the KSHV homolog of Bcl-2 which has been shown to bind peptides derived from other human Bcl-2 family members. Although only about 20% identical at the sequence level with human Bcl-2 its structure still shows the same helical fold as its human counterpart. However, a few key conserved residues, such as the tryptophans in the BH1 and BH2 regions, can still be identified by sequence alignment.^43^

In this study, our computational work makes a strong case that KSHV ORF63 is not a viral NLR homolog. In the first instance, neither NBD nor LRR domains were identified when searching for conserved domains in KSHV ORF63. Indeed critical residues, including those for the Walker A motif and the asparagine ladder, required for NBD or LRR function and formation, are missing in KSHV ORF63. Second, the secondary structure predictions are highly alpha-helical and inconsistent with that expected from an NLR protein. Third, the predicted tertiary structure of KSHV ORF63 lacks any similarity with NLR proteins. In combination, these computational analyses provide compelling evidence that KSHV ORF63 is a mainly helical protein.

KSHV ORF63 is not the first example of a protein incorrectly assigned to a particular protein fold on the basis of small regions of sequence similarity. For example, the poxviral protein A46R was originally reported to possess a TIR (Toll Interleukin-1 Receptor) fold. This was determined upon sequence conservation between the viral protein and the boxes 1, 2, and 3 motifs of TIR domains.^44^ However, determination of the structure of related viral proteins, coupled with improvements in molecular modelling approaches indicates that this protein is likely to have a Bcl2-like fold.^45^ It is clear that interpretation of sequence homology between viral and non-viral proteins is more complex than between eukaryotic species, or even eukaryotes and prokaryotes. Consequently, to help avoid incorrect predictions of protein homology or folding it is important that bioinformatic tools are used carefully and comprehensively.

**Figure 4:**
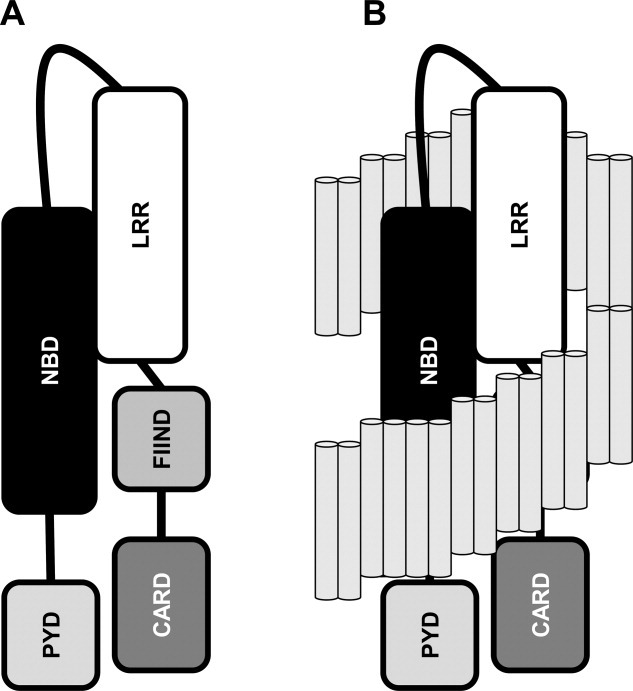
Mechanism for KSHV ORF63 mediated inhibition of inflammasome signaling. **A:** Modular domain organization of human NLRP1 in which the protein is maintained in an inactive conformation through interactions between the NBD (black) and LRR (white) domains. **B:** Inhibition of NLRP1 activation by an alpha helical rich KSHV ORF63. The KSHV ORF63 protein may wrap itself around NLRP1 with HEAT-like repeats making multiple contacts with regions of NLRP1. The tandem alpha helices that constitute a HEAT repeat are represented as light gray cylinders.

A burning question remains––how does KSHV ORF63 interfere with inflammasome signaling? Ultimately determining the precise structure, homology and mechanism of action for KSHV ORF63 will require the molecular structure to be solved. However, a working model based upon a HEAT-repeat like structure of KSHV ORF63 can be generated for the disruption of inflammasome signaling (Fig. 4). Classical HEAT repeats consist of a helical hairpin formed from two alpha helices^46^ and often function as protein–protein interaction domains. KSHV ORF63 is a large protein. In a manner analogous to the role of Cand1 in the regulation of the formation of multisubunit Cullin-dependent ubiquitin ligase signaling complexes^31^ KSHV ORF63 could conceivably wrap itself around its target NLR, coming into contact with multiple domains of the NLR protein. This could lock the NLR in an inactive conformation; interfere with co-factor recruitment; or impede conformational rearrangements required after ligand stimulation.

It is clear that although KSHV ORF63 does disrupt NLR signaling it is unlikely to be a viral NLR-homolog. This would not be unprecedented. Indeed many proteins that interact with NLR family members to regulate or inhibit activity are not NLR homologs, though in some instances they may possess some similar protein folds. These include proteins such as SGT1^47^ and Erbin^48^; as well as human Bcl-2 and Bcl-X_L_ which bind directly to NLRP1, preventing it from forming an inflammasome in response to MDP.^49^ The unstructured viral protein NS1 is also reported to block caspase-1 activation through the inflammasome, though the mechanism behind this function is unknown.^22^ To obtain the full picture of how KSHV ORF63 disrupts inflammasome signaling we will need more information on the exact regions of KSHV ORF63 and the NLR proteins that interact as well as the structure of KSHV ORF63. Together these will provide important and exciting information about the innate immune evasion strategies of KSHV.
